# PACS-2 deficiency aggravates tubular injury in diabetic kidney disease by inhibiting ER-phagy

**DOI:** 10.1038/s41419-023-06175-3

**Published:** 2023-10-04

**Authors:** Jinfei Yang, Li Li, Chenrui Li, Wei Chen, Yan Liu, Shilu Luo, Chanyue Zhao, Yachun Han, Ming Yang, Hao Zhao, Na Jiang, Yiyun Xi, Chengyuan Tang, Juan Cai, Li Xiao, Huafeng Liu, Lin Sun

**Affiliations:** 1 Department of Nephrology, the Second Xiangya Hospital of Central South University, Hunan Key Laboratory of Kidney Disease and Blood Purification, Changsha, Hunan China; 2 Key Laboratory of Prevention and Management of Chronic Kidney Disease of Zhanjiang, Institute of Nephrology, Affiliated Hospital of Guangdong Medical University, Zhanjiang, Guangdong China

**Keywords:** Diabetes complications, Nephrons, Autophagy

## Abstract

Autophagy of endoplasmic reticulum (ER-phagy) selectively removes damaged ER through autophagy-lysosome pathway, acting as an adaptive mechanism to alleviate ER stress and restore ER homeostasis. However, the role and precise mechanism of ER-phagy in tubular injury of diabetic kidney disease (DKD) remain obscure. In the present study, we demonstrated that ER-phagy of renal tubular cells was severely impaired in streptozocin (STZ)-induced diabetic mice, with a decreased expression of phosphofurin acidic cluster sorting protein 2 (PACS-2), a membrane trafficking protein which was involved in autophagy, and a reduction of family with sequence similarity 134 member B (FAM134B), one ER-phagy receptor. These changes were further aggravated in mice with proximal tubule specific knockout of *Pacs-2* gene. In vitro, transfection of HK-2 cells with PACS-2 overexpression plasmid partially improved the impairment of ER-phagy and the reduction of FAM134B, both of which were induced in high glucose ambience; while the effect was blocked by FAM134B siRNA. Mechanistically, PACS-2 interacted with and promoted the nuclear translocation of transcription factor EB (TFEB), which was reported to activate the expression of FAM134B. Collectively, these data unveiled that PACS-2 deficiency aggravates renal tubular injury in DKD via inhibiting ER-phagy through TFEB/FAM134B pathway.

## Introduction

Diabetic kidney disease (DKD) is the major microvascular complication of diabetes, and is also the most critical pathogenic factor for end-stage renal disease [1, 2]. Recent findings lead to the paradigm shift of DKD from previous “glomerulocentric” viewpoint to the current “tubulogenic” view [3, 4]. Tubulopathy was found preceded glomerular abnormalities in DKD patients and involved in the pathogenesis of DKD [5, 6]. The role of subcellular organelle injury and impaired autophagy of some subcellular organelles in tubulopathy of DKD was confirmed in our previous studies [7–9]. But the detailed mechanisms of these disturbed autophagic organelle turnover are still not fully explained. Endoplasmic reticulum (ER) homeostasis disruption is well recognized as the critical pathogenesis causing tubular cell damage and DKD progression [10, 11]. However, the role and precise mechanisms of autophagy clearance of ER (ER-phagy) in the pathogenies of diabetes-induced tubular injury remain obscure.

The ER is the central site of protein folding, processing and modification [12]. Keeping the homeostasis of ER is fundamental for the physiological function of ER, and is also essential for maintaining cellular homeostasis [13]. Disruption of ER structure and function is an important mechanism for diabetic tubulopathy [14, 15]. Recent evidence shows that the autophagy of the ER, referred to as ER-phagy, is conducive to alleviating ER stress and restoring ER homeostasis by selectively recognizing and degrading redundant and damaged ER, thereby protecting cells from unbalanced cellular homeostasis caused by excessively accumulated ER [16–19]. The identification of ER-phagy receptors, such as family with sequence similarity 134 member B (FAM134B), ensures the understanding of the physiological significance and precise mechanisms of ER-phagy in various diseases [18, 20]. However, the exploration of ER-phagy in kidney disease is inchoate recently. Jiang et al. have described that the hyperactivation of ER-phagy was involved in quantum dots-induced nephrotoxicity [21]. However, the role and regulation of ER-phagy in DKD remain unclear.

Phosphofurin acidic cluster sorting protein 2 (PACS-2) is a membrane trafficking protein [22] and is also a key regulator of mitochondria associated ER membranes (MAMs) [23]. We have previously showed that depletion of PACS-2 in diabetic mice would accelerate the progression of DKD [9], indicating a protective role of PACS-2 in tubulopathy of DKD. It has also been observed that knockdown of *Pacs-2* could significantly hinder the autophagy process [24]. Recently, the function of PACS-2 in maintaining ER homeostasis in renal tubular cells was also proved [25]. Given that PACS-2 is involved in autophagy and ER stability, we speculate that PACS-2 may ameliorate DKD tubular injury by participating in the process of ER-phagy.

In this study, we applied the previously constructed renal proximal tubular *Pacs-2* knockout (Pacs-2^ptKO^) mice to observe the impact of PACS-2 on ER-phagy, and found that PACS-2 deficiency aggravated the ER-phagy disruption in renal proximal tubular epithelial cells of diabetic mice, accompanied by excessive inflammatory response and increased fibrosis. In vitro studies demonstrated that overexpression of PACS-2 up-regulated FAM134B expression, restored ER-phagy and reduced the production of inflammatory cytokine and fibronectin (FN) in high glucose (HG)-treated HK-2 cells; while these changes could be inhibited by transfection of FAM134B siRNA. Moreover, an induction of nuclear translocation of transcription factor EB (TFEB), a transcription factor that promotes the transcription of *Fam134b* gene, by PACS-2 overexpression and a direct interaction between TFEB and PACS-2 were observed in this study. These data demonstrated for the first time that disrupted ER-phagy induced by PACS-2 deficiency plays a key role in diabetes-induced renal tubular injury.

## Materials and methods

### Primary antibodies

The following primary antibodies were used in this study: the anti-LC3B (sc-271625) was purchased from Santa Cruz Biotechnology; the anti-Collagen IV (ab6586), anti-Fibronectin (ab2413), anti-LC3β (ab192890), and anti-Collagen I (ab34710) were purchased from Abcam; the anti-Interleukin-1β (16806-1-AP), anti-CKAP4 (16686-1-AP), anti-Calnexin (10427-2-AP), anti-Interleukin-18 (60070-1-IG), anti-β-actin (60008-1-IG), anti-PACS-2 (19508-1-AP), anti-TFEB (13372-1-AP), anti-GAPDH (60004-1-IG) and anti-Lamin B1 (66095-1-IG) were purchased from Proteintech; the anti-FAM134B (83414 S) was purchased from Cell Signaling Technology; and anti-Flag (F1804) was purchased from Sigma-Aldrich.

### Mouse model

Renal proximal tubular *Pacs-2* knockout (Pacs-2^ptKO^) mice and the littermate control (Pacs-2^ctrl^) mice were generated by crossing Pacs-2^fl/fl^ mice (C57BL/6 J background) with Ggt1-Cre mice (mixed Balb/cJ/C57BL/6 background), and were identified as described previously [9]. Determination of sample size was based on our previous observations. Male mice of both genotypes were then randomly divided into the following groups: a streptozocin (STZ) treated group and a citrate buffer treated group. They were given an intraperitoneal injection of STZ (50 mg/kg body weight in citrate buffer, pH 4.5; Sigma-Aldrich) or citrate buffer for 5 days at the age of 6–8 weeks as described before [9]. One week after the injection, mice with blood glucose levels >16.7 mmol/L were included into the experiment and fed for additional 12 weeks. The body weight and the blood glucose were monitored every 2 weeks. The urine samples were harvested before the mice were sacrificed. The urine albumin concentration and creatinine levels were measured as described previously [9]. And kidney tissues were collected for further experiments. All procedures were approved by the Animal Experimentation Ethics Committee of Second Xiangya Hospital of Central South University. No blinding was performed in tests.

### Histopathological analysis of the kidney

Mice kidney tissues were fixed with 4% paraformaldehyde and embedded in paraffin. Four-μm-thick kidney sections were stained with hematoxylin-eosin (HE), periodic acid-schiff (PAS) and Masson. Tubular interstitial damage was evaluated as previously described [9].

### Real-time quantitative PCR

Total RNA was extracted from kidney cortices and HK-2 cells using RNAex Pro Reagent (Accurate Biology) according to the manufacturer’s protocol. Then, RNA was reverse transcripted to cDNA using Evo M-MLV RT Kit with gDNA Clean for qPCR (Accurate Biology). Subsequently, real-time quantitative PCR was performed using SYBR® Green Premix Pro Taq HS qPCR Kit (Accurate Biology). Primers used in this study are as follows: NGAL (mouse): sense: 5ʹ-GCAGGTGGTACGTTGTGGG-3ʹ, antisense: 5ʹ-CTCTTGTAGCTCATAGATGGTGC-3ʹ; β-actin (mouse): sense: 5ʹ-GTGACGTTGACATCCGTAAAGA-3ʹ, antisense: 5ʹ-GCCGGACTCATCGTACTCC-3ʹ; FAM134B (mouse): sense: 5ʹ-AAACAGCAGAGTCCTGGCAAG-3ʹ, antisense: 5ʹ-AGGTAGCTGAGTATGACCCCA-3ʹ; TEX264 (mouse): sense: 5ʹ-TACCTTCCCTTACACCACCC-3ʹ, antisense: 5ʹ-GTGGGCACATGAAATGGATCT-3ʹ; CALCOCO1 (mouse): sense: 5ʹ-AACCAGGAGCCCAACTCTAC-3ʹ, antisense: 5ʹ-GCCTTAGGGACAACCAACAGG-3ʹ; CCPG1 (mouse): sense: 5ʹ-AGGAATCCAGTAGCGATGACA-3ʹ, antisense: 5ʹ-CTAAAGTGGCGTTTACTTGGCT-3ʹ; SEC62 (mouse): sense: 5ʹ-TGATTGCAGTAATAGCAGCCAC-3ʹ, antisense: 5ʹ-GCCCACACTGAGGTAATAAACAC-3ʹ; RTN3 (mouse): sense: 5ʹ-CTGATTTTCTGGCGAGATGTGA-3ʹ, antisense: 5ʹ-GGATGAGGTAAGAGACCACACT-3ʹ; ATL3 (mouse): 5ʹ-AGTGCAGGTTGTTTTGGTTCA-3ʹ, antisense: 5ʹ-AGCCACTGATACTACCACCAC-3ʹ; MCP-1 (human): sense: 5ʹ-CAGCCAGATGCAATCAATGCC-3ʹ, antisense: 5ʹ-TGGAATCCTGAACCCACTTCT-3ʹ; β-actin (human): sense: 5ʹ-CCTGGCACCCAGCACAAT-3ʹ, antisense: 5ʹ-GGGCCGGACTCGTCATAC-3ʹ. Relative mRNA expression levels above were calculated with the 2^-ΔΔCt^ method and normalized to that of β-actin.

### Transcriptome analysis

Total RNA from the kidney cortices of Pacs-2^ptKO^ and Pacs-2^ctrl^ mice was extracted and purified using miRNeasy Mini Kit (QIAGEN) according to the manufacturer’s instructions. Quality control of samples and subsequent transcriptome study were performed at Wuhan SeqHealth Tech Co., Ltd. Gene Ontology (GO) enrichment analysis was conducted by online function annotation tool DAVID (https://david.ncifcrf.gov/, version 6.8) [26, 27] and visualized by a data visualization website (http://www.bioinformatics.com.cn/).

### Transmission electron microscopy of kidney tissue

After rapid isolation, fresh kidney tissues (1 × 1 × 1 mm^3^) were immersed in 1% osmium tetroxide solution for 1 h after fixed with 2.5% glutaraldehyde overnight at 4 °C. Then the samples were dehydrated in ethanol and embedded in Epon 812. Ultrathin sections were prepared and stained with uranyl acetate and lead citrate as previously described [28], followed by observation of double-membrane structure autophagosome encapsulating ER using transmission electron microscopy.

### Immunofluorescence of kidney tissue

Briefly, four-μm-thick paraffin-embedded kidney tissue sections were deparaffined, rehydrated, and antigen repaired as described previously [9]. Next, the sections were permeabilized with 0.3% TritonX-100 for 20 min and then blocked with 5% BSA in PBS buffer for 60 min at room temperature. After incubated with primary antibodies at 4 °C overnight, the sections were washed with PBS and incubated with Alexa Fluor-488- or Alexa Fluor-594-labeled secondary antibodies (Abcam) at room temperature for 1 h. DAPI (SouthernBiotech) was used to stain nuclei.

### Western blot assay

Proteins of kidney cortices or cultured cells were extracted using RIPA buffer (CWBIO) supplemented with protease inhibitors and phosphatase inhibitors (CWBIO). The protein concentrations were then determined by using BCA Protein Assay Kit (Thermo Fisher Scientific). After 5× SDS loading buffer (CWBIO) was added and boiled for 10 min, equal amounts of proteins were subjected to SDS-PAGE and transferred onto polyvinylidene fluoride membranes. Then the membranes were blocked at room temperature for 1 h and subsequently incubated with primary antibodies at 4 °C overnight and corresponding secondary antibodies (Abcam) at room temperature for 40 min. The bands were detected using an enhanced chemiluminescence kit (Thermo Fisher Scientific) by Tanon 5200 Multi instrument (Tanon Instruments). Quantification of the band intensity was measured by ImageJ software (National Institutes of Health).

### Immunohistochemistry of kidney tissue

The kidney sections were deparaffined, rehydrated, and antigen repaired. After blocking endogenous peroxidase activity and nonspecific binding sites with Peroxide Blocking Solution and 5% BSA, the sections were incubated with primary antibodies at 4 °C overnight and corresponding secondary antibodies conjugated with peroxidase at room temperature. Then the sections were treated with diaminobenzidine, and stained with hematoxylin. Nikon microscope was used for image acquisition, and quantification of protein expression level was determined by Image-Pro Plus 6.0 (Media Cybernetics).

### Nephroseq analysis

To investigate the expression of ER-phagy receptors in renal tubulointerstitium of patients with diabetic nephropathy (DN) and health controls (HC), we mined the Nephroseq database (https://www.nephroseq.org/resource/login.html, V5), a free platform containing publicly available gene expression profiles related to renal diseases. The dataset of the Ju CKD TubInt study [29], which contains 17 tubulointerstitium samples of patients of DKD and 31 samples of healthy living donors, as well as Schmid Diabetes TubInt dataset [30] and Woroniecka Diabetes TubInt dataset [31] were used to estimate the differences of ER-phagy receptors expression between DKD and healthy samples. Based on the above datasets, the heat map and boxplot graphs of ER-phagy receptors were further visualized by an online tool (http://www.bioinformatics.com.cn/) and GraphPad Prism 8.0, respectively.

### Cell culture and treatments

Human proximal tubular cell line (HK-2) was purchased from ATCC and cultured as previously described [7] and was exposed to medium containing different concentrations of d-glucose (5 mM or 30 mM). For overexpression of PACS-2, cells were transfected with pcDNA3 hPACS-2 flag plasmid (kindly provided by Professor Gary Thomas, Department of Microbiology and Molecular Genetics, University of Pittsburgh, Pittsburgh, PA) using Lipofectamine 3000 (Invitrogen) according to the manufacturer’s instructions. And for gene disruption, FAM134B siRNA was transfected into HK-2 cells using Lipofectamine 3000 (Invitrogen).

### Cell immunofluorescence

HK-2 cells were seeded onto confocal dishes and cultured with different treatments. After washing with PBS buffer, cells were fixed, permeabilized and blocked as previously described [28]. Then the cells were incubated with primary antibodies against Calnexin (1:200, Proteintech, 10427-2-ap) and LC3β (1:200, Santa Cruz Biotechnology, sc-271625) at 4 °C overnight. Subsequently, cells were incubated with Alexa Fluor® 594-conjugated goat anti-rabbit and Alexa Fluor® 488-conjugated goat anti-mouse antibodies after washing, followed by staining the nuclei with DAPI. LSM 780 META laser scanning microscopy (Zeiss) was then used for image acquisition.

### Cell transmission electron microscopy

After intervention, pour off medium and replace with 2.5% glutaraldehyde at room temperature for fixation in dark for 5 min. Then scrape the cells with a rubber scraper gently and centrifuge at 3000 rpm for 5 min to pellet the cells. Remove supernatant, add 2.5% glutaraldehyde for fixation for 30 min at room temperature in dark and fix at 4 °C overnight. After centrifugation, discard the supernatant and wash the precipitation in PBS for 3 times. Then the precipitation was suspended with forceps and wrapped in agarose. Post fix the samples with 1% osmic acid avoiding light for 2 h at room temperature, then wash it with PBS for 3 times. After dehydration in ethanol, the samples were embedded in Epon 812 and kept in 37 °C oven overnight before polymerization. Then cut resin blocks to ultrathin sections. Using 2% uranium acetate and subsequent 2.6% lead citrate to stain. Observe ultrastructure of HK-2 cells and capture images under transmission electron microscopy.

### Isolation of nuclear and cytoplasmic protein

The nuclear protein and cytoplasmic protein of HK-2 cells were extracted using Nuclear and Cytoplasmic protein Extraction Kit (Beyotime) according to the manufacturer’s instructions. Briefly, HK-2 cells were scraped and collected from culture dishes first. Then the cell pellet was put under vortex movement for 5 s after adding cytoplasmic protein extraction agent A that mixed with PMSF. After incubated on ice for 15 min, cytoplasmic protein extraction agent B was added, and the mixture was put under vortex movement for another 5 s and incubated on ice for 1 min. Subsequently, the samples were centrifuged at 16000 × *g* at 4 °C for 5 min. The supernatant was cytoplasmic protein fraction. The left precipitation was centrifuged again, and the residual supernatant was discarded as far as possible. The precipitation was then added with nuclear protein extraction agent supplemented with PMSF and suspended under vortex movement every 1 min for 30 s. After 30 min, the suspension was centrifuged at 16000 × *g* at 4 °C for 10 min and the obtained supernatant was nuclear protein fraction. The subsequent Western blot steps are as described above.

### Protein-protein molecular docking

Crystal structures of TFEB and PACS-2 were obtained from AlphaFold Protein Structure Database [32, 33]. Then the PDB format files of these proteins were imported into GRAMM-X Protein-Protein Docking Web Server v.1.2.0 [34, 35]. The exported 10 potential interaction modes were visualized using PyMOL (Version 2.4.0).

### Immunoprecipitation

HK-2 cells were co-transfected with flag-tagged PACS-2 plasmid and TFEB plasmid or transfected with TFEB plasmid but without PACS-2 plasmid for 48 h. Cells were then lysed with immunoprecipitation lysis buffer (Thermo Fisher Scientific) mixed with protease inhibitors and phosphatase inhibitors and incubated with anti-flag magnetic beads (Bimake) at 4 °C overnight. The precipitate was subsequently used for Western blot analysis with primary antibodies against flag (1:5000, Sigma-Aldrich, F1804) and TFEB (1:1000, Proteintech, 13372-1-AP).

### Statistical analysis

The experimental data was presented as mean ± SD unless otherwise specified. Statistical analysis was implemented using GraphPad Prism (Version 8.0) and SPSS (Version 23.0). Student t-test and one-way analysis of variance (ANOVA) with Tukey’s test post hoc analysis were used to compare the differences between groups. And Pearson’s correlation analysis was used for the correlation analysis between two variables. *P* values < 0.05 were considered statistically significant.

## Results

### *Pacs-2* gene ablation worsened renal tubular injury, interstitial inflammation and fibrosis in diabetic mice

**Fig. 1:**
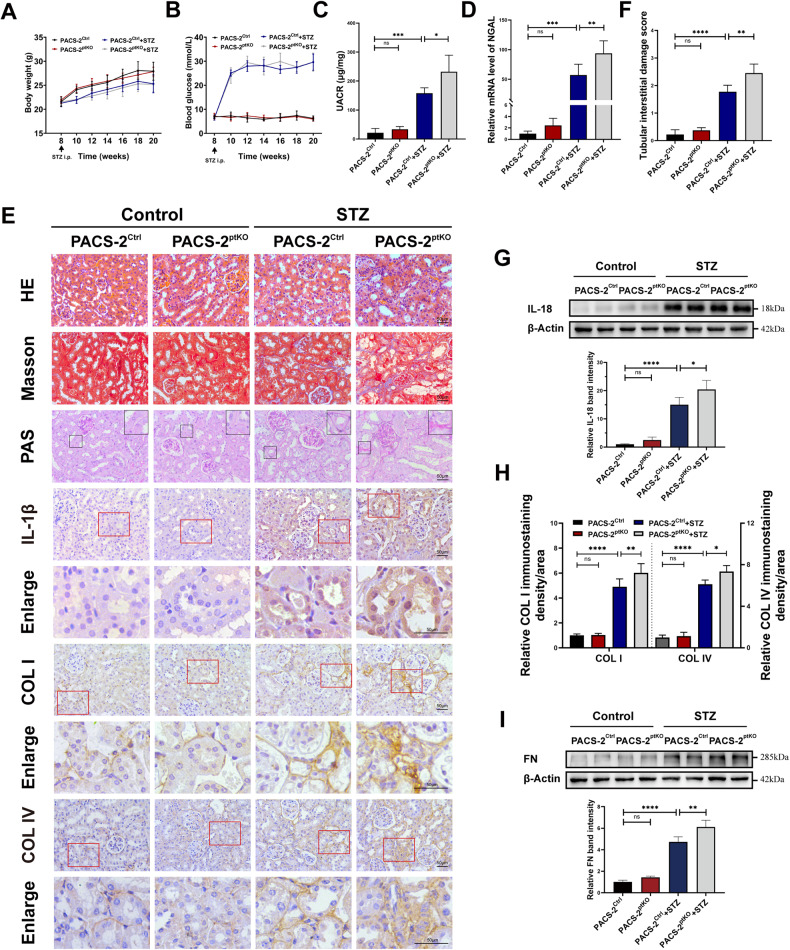
Proximal tubule-specific *Pacs*-2 deficiency worsened renal tubular injury in STZ-induced diabetic mice. **A** Body weight; (**B**) blood glucose levels; (**C**) urinary albumin-to-creatinine ratio (UACR); and (**D**) Real-time quantitative PCR showing the mRNA levels of NGAL in kidney cortices of each group; (**E**) Haematoxylin-eosin (HE) staining, Masson staining, Periodic Acid Schiff (PAS) staining, immunohistochemical (IHC) analysis of IL-1β, COL I and COL IV in each group. Scale bar: 50 μm. **F** Quantification of tubular interstitial damage score of kidneys. **G** Western blot analysis and quantification of the IL-18 protein expression in each group. **H** Semi-quantification of the IHC analysis of COL I and COL IV expression. **I** Western blot analysis and quantification of the FN levels. Values are presented as mean ± SD, ^*^P < 0.05, ^**^P < 0.01, ^***^P < 0.001, ^****^P < 0.0001, ns, not significant. *n* = 4. i.p. intraperitoneal.

To gain a better perspective on the role of PACS-2 in the progression of DKD, we generated mice with proximal tubule specific knockout of *Pacs-2* gene (Pacs-2^ptKO^) and induced diabetic states by injecting STZ intraperitoneally as described previously [9]. As shown in Fig. 1A, B, both STZ-treated groups exhibited hyperglycemia, with a lower body weight than that of the non-STZ-injected groups, indicating successful construction of diabetes model. Little differences of body weight and blood glucose were observed between diabetic Pacs-2^Ctrl^ mice and diabetic Pacs-2^ptKO^ mice (Fig. 1A, B). However, a significant elevation of the urinary albumin-to-creatinine ratio (UACR) was found in diabetic Pacs-2^ptKO^ group when compared to diabetic Pacs-2^Ctrl^ mice (Fig. 1C). Similar changes were also spotted in the qPCR detection of neutrophil gelatinase-associated lipocalin (NGAL), a biomarker of tubulopathy (Fig. 1D).

Pronounced histological differences were also detected. As shown in HE, Masson and PAS staining, both diabetic groups displayed notable loss of brush borders in proximal tubules, interstitial inflammatory cell infiltration, tubular basement-membrane thickening, and tubulointerstitial fibrosis, especially the diabetic Pacs-2^ptKO^ group (Fig. 1E, F). Besides, immunostaining of interleukin-1 beta (IL-1β) indicated an obvious increase of inflammatory cytokine when comparing diabetic Pacs-2^Ctrl^ mice to nondiabetic Pacs-2^Ctrl^ mice, and a more significant increase was found in diabetic Pacs-2^ptKO^ mice (Fig. 1E). The increased expression level of inflammatory cytokines in diabetic Pacs-2^ptKO^ mice was also confirmed by Western blot analysis of interleukin-18 (IL-18) (Fig. 1G). Additionally, tubulointerstitial fibrosis in diabetic mice was also substantiated by immunostaining of collagen type IV (COL IV) and collagen type I (COL I), and the positive staining was further increased in diabetic Pacs-2^ptKO^ mice (Fig. 1E, H). Western blot analysis of FN also revealed a significantly increased expression of extracellular matrix protein in diabetic mice. And this change was further aggravated in diabetic Pacs-2^ptKO^ mice (Fig. 1I). Taken together, these observations support a latent reno-protective role of PACS-2 in diabetic tubulointerstitial injury.

### *Pacs-2* gene deficiency blocked ER-phagy in the renal tubules of diabetic mice

**Fig. 2:**
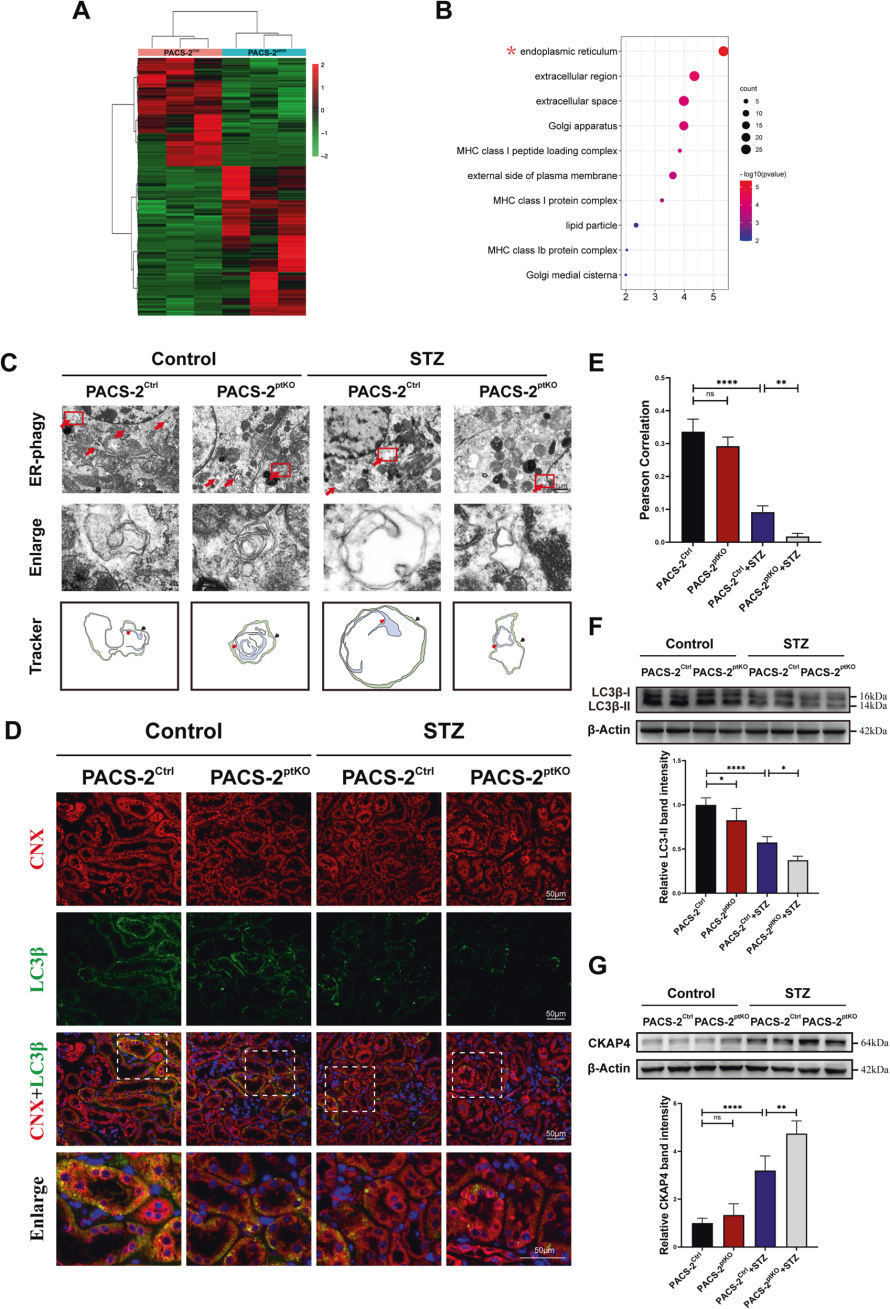
Proximal tubule-specific *Pacs-2* gene deficiency blocked ER-phagy in the renal tubular cells of diabetic mice. **A** Heat map of differentially expressed genes (DEGs) in kidney cortices of Pacs-2^Ctrl^ mice and Pacs-2^ptKO^ mice (*n* = 3). Red represents up-regulation, green represents down-regulation, and black represents no change. **B** Bubble plot of the top 10 enriched cellular components of DEGs by Gene Ontology enrichment analysis. **C** Transmission electron microscopy images of renal tubular cells in each group (first panel, red arrows indicate ER-phagy structures), magnified figures of ER-phagy found in the first panel (second panel), and localization patterns of autophagosomes of ER-phagy (third panel, the red arrows represent encapsulated ER components, and the black arrows represent double-membrane autophagic vacuoles). Scale bar: 1 μm. **D** Representative immunofluorescence images of LC3β (green) and CNX (red) in kidney tissues from each group. Nuclei were counterstained with DAPI (blue). Scale bar: 50 μm. **E** Quantification of the colocalization levels between CNX and LC3β by Pearson’s correlation coefficient. **F**, **G** Western blot analyses and quantifications of the protein levels of LC3β-II and CKAP4. Values are presented as mean ± SD, ^*^P < 0.05, ^**^P < 0.01, ^****^P < 0.0001, ns, not significant. Unless otherwise specified, *n* = 4.

We then investigated the mechanism by which PACS-2 plays a protective role in DKD through transcriptomics analysis. As shown in the heat map, a total of 219 differentially expressed genes (DEGs) were obtained through transcriptome analysis of renal cortices from Pacs-2^Ctrl^ mice and Pacs-2^ptKO^ mice (Fig. 2A). Gene Ontology analysis was carried out subsequently, and the result revealed that these DEGs were mainly enriched in ER according to cellular component analysis, indicating that *Pacs-2* gene ablation in proximal tubule would affect ER remarkably **(**Fig. 2B). Transmission electron microscopy analysis was then conducted to observe the ultrastructural changes of renal tubular cells in each group, especially the changes of ER. As shown in Fig. 2C, ER-phagosomes, a phenomenon that ER was encapsulated by double-membrane autophagosomes, were detected in the renal tubular cells of all groups. However, less ER-phagosomes were presented in diabetic mice, indicating that ER-phagy deficiency might be closely correlated with the pathological injury of DKD; and a more significant decrease was observed in diabetic Pacs-2^ptKO^ mice. Co-staining for LC3β and calnexin (CNX), an autophagosome marker and an ER membrane protein, respectively, was used to delineate the activation of ER-phagy. The colocalization of punctate LC3β and CNX was decreased in tubules from diabetic Pacs-2^Ctrl^ mice, and was further reduced in diabetic Pacs-2^ptKO^ mice (Fig. 2D, E). Similar alterations were found in the detection of LC3β-II expression level through Western blot analysis (Fig. 2F). The level of ER-phagy was further evaluated by determining the clearance of ER resident protein cytoskeleton-associated protein 4 (CKAP4). In accordance with the above findings, the degradation of ER was blocked in the kidney of diabetic Pacs-2^Ctrl^ mice, and was further accentuated in diabetic Pacs-2^ptKO^ mice, as a more remarkable increase of CKAP4 was detected in the diabetic Pacs-2^ptKO^ mice (Fig. 2G).

### *Pacs-2* gene deficiency inhibited the expression of ER-phagy receptor FAM134B

**Fig. 3:**
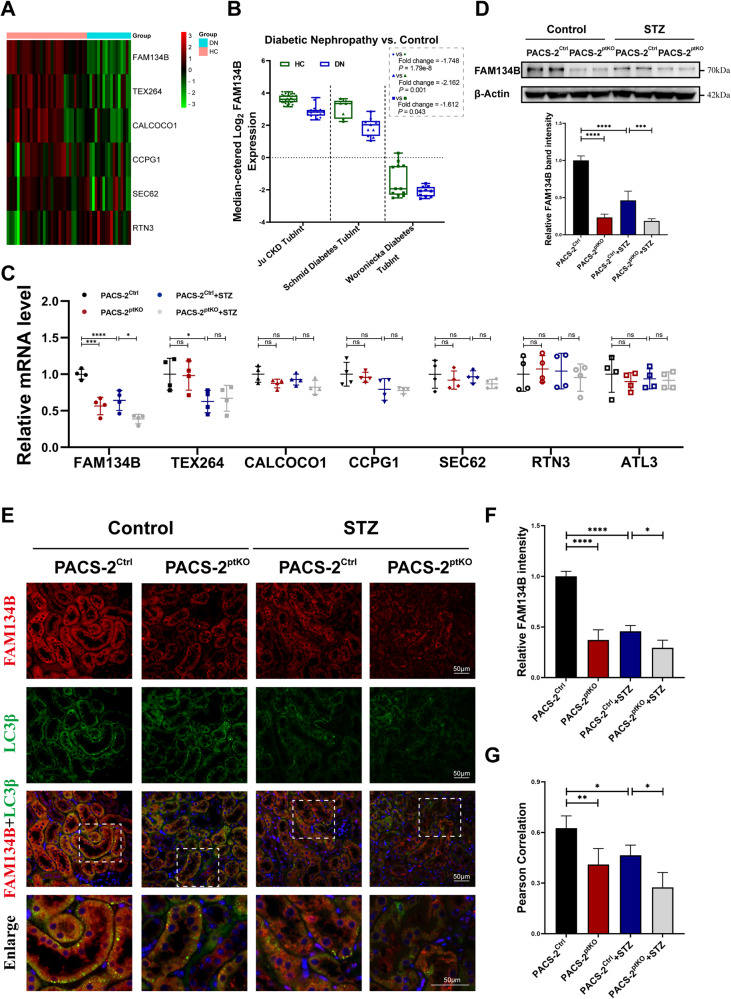
Proximal tubular cells-specific *Pacs-2* deficiency decreased the expression of ER-phagy receptor FAM134B. **A** Heat map of ER-phagy receptors in Ju CKD TubInt transcriptome dataset mined from Nephroseq database. Red represents up-regulation, green represents down-regulation, and black represents no change. **B** Comparisons of FAM134B expression levels between health control group and diabetic nephropathy group in Ju CKD TubInt, Schmid Diabetes TubInt, and Woroniecka Diabetes TubInt transcriptome datasets from Nephroseq database. Values are represented as median ± quartiles. **C** Real-time quantitative PCR analyses of the mRNA levels of ER-phagy receptors FAM134B, TEX264, CALCOCO1, CCPG1, SEC62, RTN3 and ATL3 in kidney cortices of different groups. **D** Western blot analysis and quantification of the FAM134B protein expression levels. **E** Representative immunofluorescence images of LC3β (green) and FAM134B (red) in kidney tissues from each group. Nuclei were counterstained by DAPI (blue). Scale bar: 50 μm. **F** Semi-quantification of the immunostaining analysis of FAM134B. **G** Quantification of the colocalization levels between FAM134B and LC3β by Pearson’s correlation coefficient. Values are presented as mean ± SD unless otherwise specified, ^*^P < 0.05, ^**^P < 0.01, ^***^P < 0.001, ^****^P < 0.0001, ns, not significant. *n* = 4. DN diabetic nephropathy, HC health controls.

As ER-phagy receptors that located on ER or recruited to ER play a central role in the ER-phagy process [19], we then mined the expression data of ER-phagy receptors in renal tubulointerstitium of healthy controls and DKD patients in the Nephroseq online database. The expression data of ER-phagy receptors in Ju CKD TubInt dataset was summarized in Fig. 3A. As shown in the heat map, when compared to healthy controls, the mRNA levels of FAM134B, TEX264 and CALCOCO1 were much lower in DKD patients, while no significant differences were found for other receptors such as CCPG1, SEC62 or RTN3. Detailed analyses of the most significantly changed receptor, FAM134B, were presented in Fig. 3B. The results from different datasets were consistent with each other. Next, we evaluated the mRNA levels of ER-phagy receptors in diabetic mice and nondiabetic mice with or without *Pacs-2* gene deficiency. As shown in Fig. 3C, when compared to nondiabetic Pacs-2^Ctrl^ mice, a notable decrease of the mRNA levels of FAM134B and TEX264 in the kidney of diabetic Pacs-2^Ctrl^ mice was observed. Further, we found that the mRNA level of FAM134B was also reduced in nondiabetic Pacs-2^ptKO^ mice when compared to nondiabetic Pacs-2^Ctrl^ mice. And the relative mRNA level was further lower in the diabetic Pacs-2^ptKO^ mice than in the diabetic Pacs-2^Ctrl^ mice. While no such alterations were observed for TEX264 in Pacs-2^ptKO^ mice with or without STZ injection. The comparisons of other receptors among groups showed no remarkable differences. Since the above results indicated that *Pacs-2* gene deficiency might influence the expression of FAM134B at the transcription level, we then detected the abundance of FAM134B in renal cortices utilizing Western blot and immunofluorescence. As expected, the expression level of FAM134B in diabetic mice was lower than in nondiabetic mice, and *Pacs-2* gene deficiency decreased the abundance of FAM134B no matter in diabetic mice or in nondiabetic mice (Fig. 3D–F). Since FAM134B mediates ER-phagy through anchoring ER to autophagic vesicles by binding with LC3β, co-staining of FAM134B and LC3β was performed. Similarly, colocalization between punctate LC3β and FAM134B was highly reduced in tubules from Pacs-2^ptKO^ mice and diabetic Pacs-2^Ctrl^ mice when comparing to nondiabetic Pacs-2^Ctrl^ mice, and was further decreased in diabetic Pacs-2^ptKO^ mice (Fig. 3E, G).

### PACS-2 reversed the inflammation and fibrosis by activating FAM134B induced ER-phagy in HK-2 cells exposed to HG ambience

**Fig. 4:**
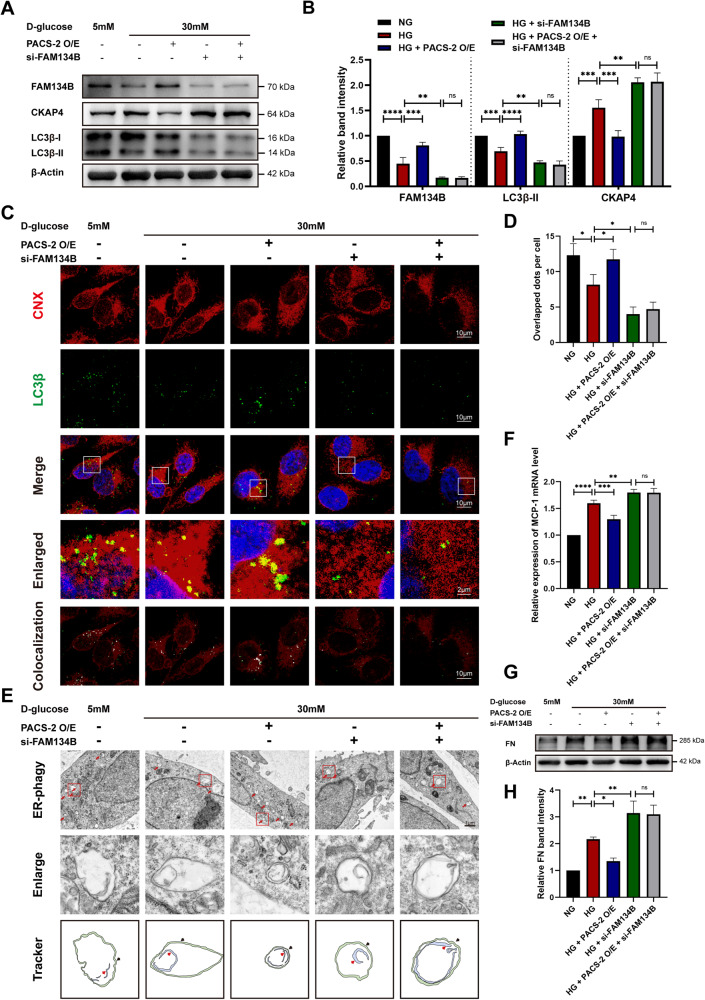
Overexpression of PACS-2 reversed high glucose (HG) induced inflammation and fibrosis in HK-2 cells by activating FAM134B induced ER-phagy. **A**, **B** Western blot analyses and quantifications of the protein expression levels of FAM134B, CKAP4 and LC3β-II in HK-2 cells treated as indicator. **C** Confocal images of CNX (red) and LC3β (green) in HK-2 cells. Nuclei were counterstained with DAPI (blue). Overlap was analyzed by the colocalization plugin (ImageJ) and is shown in white. **D** Quantification of the overlap of CNX and punctate LC3β. **E** Transmission electron microscopy images of HK-2 cells in each group (first panel, red arrows indicate ER-phagy structures), magnified figures of ER-phagy found in the first panel (second panel), and localization patterns of autophagosomes of ER-phagy (third panel, the red arrows represent encapsulated ER components, and the black arrows represent double-membrane autophagic vacuoles). Scale bar: 1 μm. **F** Real-time quantitative PCR analysis of mRNA expression of MCP-1 in HK-2 cells. **G**, **H** Western blot analysis and quantification of FN expression. Values are presented as mean ± SD, ^*^P < 0.05, ^**^P < 0.01, ^***^P < 0.001, ^****^P < 0.0001, ns, not significant. *n* = 3. NG normal glucose, HG high glucose, O/E overexpression, si-FAM134B FAM134B siRNA.

The above in vivo results hint a possibility that the deficiency of PACS-2 protein might participate in DKD tubulointerstitial damage by hindering FAM134B mediated ER-phagy. To further determine whether FAM134B is the intermediate molecule of PACS-2 participated ER-phagy process, we assessed ER-phagy level after manipulating the protein expression of FAM134B and PACS-2 alone or simultaneously in vitro. As shown in Fig. 4A, B, the expression of FAM134B and LC3β-II were down-regulated after cultured with HG when compared to HK-2 cells cultured with normal glucose (NG), while overexpression of PACS-2 with plasmid reversed these changes. In contrast, inhibiting FAM134B expression via siRNA further reduced LC3β-II expression. And inducing PACS-2 expression did not reverse the decreased expression of LC3β-II in FAM134B knockdown cells under HG ambience. Further, the degradation of substrate was also estimated by determining the expression level of CKAP4. Results showed that HG treatment increased CKAP4 expression. The effect was enhanced by transfection with FAM134B siRNA but abrogated by overexpressing PACS-2. However, the degradation of CKAP4 that was induced by PACS-2 overexpression was blocked by FAM134B siRNA transfection.

The colocalization between punctate LC3β and CNX further convinced that the formation of ER-phagosomes in HK-2 cells was largely reduced after cultured with HG, and overexpressing PACS-2 restored ER-phagy, while inhibiting FAM134B expression in cells cultured with HG further reduced the colocalization (Fig. 4C, D). However, overexpressing PACS-2 could not reverse the down-regulated ER-phagosome formation that was caused by FAM134B siRNA in HG-treated HK-2 cells (Fig. 4C, D). Transmission electron microscopy was also used to observe the changes of ER-phagy. As shown in Fig. 4E, less ER-phagosomes were detected in HG group when compared to NG group, and deficiency of FAM134B further accentuated the change. However, enhancing PACS-2 expression displayed an improvement effect in HG cultured cells, while the effect was blocked by FAM134B siRNA transfection (Fig. 4E). These results indicate that PACS-2 activates ER-phagy by regulating FAM134B expression in HK-2 cells.

We then tested proinflammatory cytokine and fibrosis factor expressions in each group. Notably increased levels of monocyte chemoattractant protein-1 (MCP-1) mRNA, as well as FN protein were found in the HG-cultured HK-2 cells when compared to HK-2 cells cultured under NG condition. Up-regulating PACS-2 reversed these changes induced by HG ambience, while suppressing FAM134B expression further increased MCP-1 expression and FN level (Fig. 4F–H). Besides, overexpression of PACS-2 could not reverse the high expression of MCP-1 and FN caused by FAM134B siRNA transfection in HG treated cells (Fig. 4F–H).

### PACS-2 promoted FAM134B expression by reversing HG inhibited TFEB nuclear translocation in HK-2 cells

**Fig. 5:**
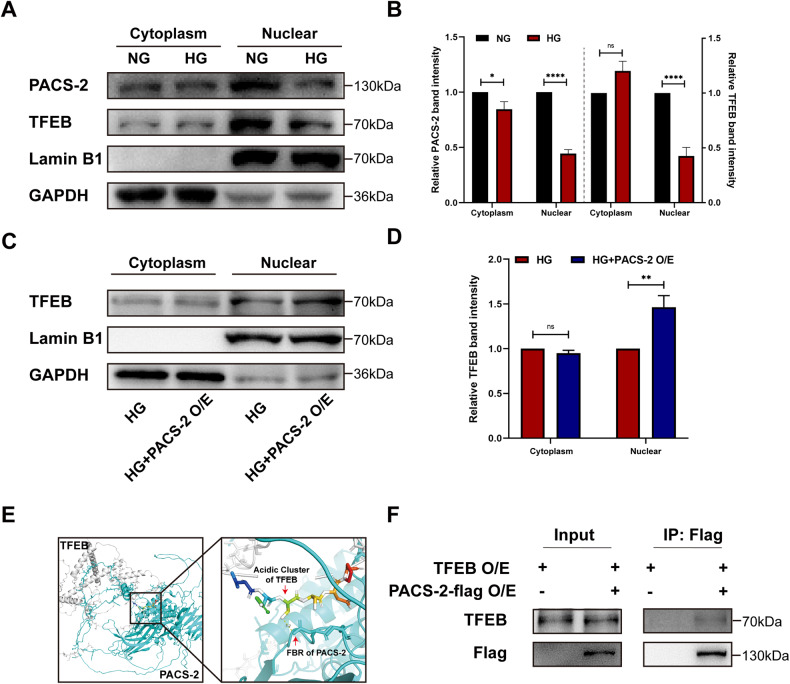
Overexpression of PACS-2 reversed HG inhibited TFEB nuclear translocation in HK-2 cells. **A**, **B** Western blot analyses and quantifications of PACS-2 and TFEB expressions in the cytoplasm fractions and the nuclear fractions isolated from HK-2 cells exposed to NG or HG. **C**, **D** Western blot analysis and quantification of TFEB expression in the cytoplasm fractions and the nuclear fractions isolated from HK-2 cells exposed to HG with or without PACS-2 overexpression. **E** The predicted interaction mode between TFEB and PACS-2. **F** Co-immunoprecipitation analysis of PACS-2-flag and TFEB in HK-2 cells co-overexpressing TFEB and PACS-2-flag or overexpressing TFEB alone by using anti-flag magnetic beads. *n* = 3. NG normal glucose, HG high glucose, O/E overexpression, FBR furin binding region.

The transcriptional regulation of FAM134B was controlled by activated transcription factor TFEB [36]. After entering nucleus, TFEB binds to a coordinated lysosomal expression and regulation (CLEAR) site that is located in the third intron of the *Fam134b* gene, facilitating *Fam134*b gene transcription [36]. Given that PACS-2 has a nuclear localization signal domain [22], we wonder if PACS-2 could promote nuclear translocation of TFEB in HK-2 cells, as membrane trafficking is a major function of PACS-2 [22]. We then isolated the nuclear fraction from HK-2 cells cultured with NG or with HG. Western blot assays showed that the abundances of PACS-2 and TFEB in nucleus were reduced in HG-cultured cells when compared to NG-cultured cells, with the nucleus-specific marker Lamin B1 serving as a loading control (Fig. 5A, B). Further, we determined whether up-regulating PACS-2 by plasmid would affect nuclear TFEB expression. As shown in Fig. 5C, D, PACS-2 overexpression enhanced the localization of TFEB in nucleus that was inhibited by HG.

Canonical acidic cluster of cargo proteins was necessary for membrane trafficking of PACS-2 [22]. By analyzing the amino acid sequence of TFEB protein, we found that TFEB also contained acidic cluster sequences that can be recognized by PACS-2 cargo binding region, suggesting that TFEB and PACS-2 may bind to each other. To test the hypothesis, an online protein-protein interaction tool, GRAMM-X protein docking server, was applied to predict the interaction between PACS-2 and TFEB. By reviewing 10 exported docking results, we found that the polar bond between these two molecules in one docking mode was just between the cargo binding region of PACS-2 and the acidic cluster of TFEB (Fig. 5E). Co-immunoprecipitation followed by Western blot assay was performed in HK-2 cells co-overexpressing TFEB and PACS-2-flag, and the results showed TFEB co-precipitated with PACS-2 (Fig. 5F), indicating an interaction between PACS-2 and TFEB. To summarize, these observations indicate that PACS-2 could bind with TFEB and promote TFEB nuclear translocation in HK-2 cells.

## Discussion

In the present study, we demonstrated that proximal tubule-specific *Pacs-2* gene knockout would aggravate ER-phagy disruption, tubulointerstitial inflammation, and tubulointerstitial fibrosis in STZ-induced diabetic mice, and that PACS-2 activated ER-phagy through upregulating the expression of ER-phagy receptor FAM134B by promoting TFEB nuclear translocation. To our knowledge, this is the first report on the role of PACS-2 in maintaining ER-phagy through the TFEB/FAM134B axis in diabetic tubulopathy.

The selective autophagy, such as mitophagy, ER-phagy, pexophagy and lipophagy, plays an important role in the maintenance of cellular homeostasis [17, 37–40]. We previously found that selective autophagy dysfunction, such as the deficiency of mitophagy and lipophagy, was involved in tubular injury in DKD [28, 41]. ER-phagy is a newly discovered selective autophagic process in recent years [16] and plays a key role in the dynamic remodeling and homeostatic maintenance of ER [16, 42]. Under stress conditions, ER-phagy promotes the effective degradation of misfolded proteins accumulated in the ER lumen, thus ensuring the maintenance of ER function and the quality of newly synthesized proteins [16, 42]. Currently, the importance of ER-phagy in the pathogenesis of different diseases is gradually being recognized. A study has reported that ER-phagy could remove misfolded proinsulin aggregates in insulinoma cells and play a protective role in mutant INS-gene-induced diabetes of youth [43]. In this study, we linked impaired ER-phagy to pathological changes of proximal tubules in diabetic mice, providing a new insight into the understanding of tubular injury in DKD.

PACS-2 is mainly expressed in tubular epithelial cells in the kidney [9, 22]. In our earlier research, we observed a significant downregulation of PACS-2 expression in the renal tubules of both DKD patients and mice, revealing a potential link between PACS-2 and DKD tubulopathy [9]. Previous study has found that HG induced downregulation of PACS-2 protein and upregulation of ER stress-related proteins, while overexpressing PACS-2 could improve ER stress and apoptosis in HG-treated HK-2 cells [25]. ER stress can accelerate DKD progression through aggravating inflammation via multiple mechanisms [44, 45]. Mei Xue et al. found that PACS-2 alleviated ER stress and improved tubular epithelial cell apoptosis and renal fibrosis in DKD mice by maintaining the integrity of MAMs, thus delaying DKD progression [25]. However, ER-phagy, as a critical mechanism that re-set ER to pre-stress condition, was not explored in that study. Here, we showed that proximal tubule-specific knockout of *Pacs-2* gene further elevated the UACR level and exacerbated inflammation, fibrosis and ER-phagy disorder in renal tissues of DKD mice (Fig. 1 & Fig. 2). It suggested that PACS-2 deficiency may exert detrimental effects on tubulointerstitial inflammation and fibrosis in DKD through regulating ER-phagy.

ER-phagy is regulated by the expression and the oligomerization degree of autophagic receptors on the ER membrane [21, 43, 46]. Several ER-phagy receptors on the ER membranes have been identified, such as FAM134B, CCPG1, and RTN3L [47, 48]. These ER-phagy receptors bind to LC3β through an LC3-interacting region in the cytoplasm, and mediate ER sequestration into the autophagosomes [19, 49]. FAM134B is the earliest identified and most studied ER-phagy receptor in mammals, mediating autophagic degradation of ER [17, 19, 47], and its changes in expression or oligomerization are closely associated with tumorigenesis, viral infections, and neurological diseases [46, 50, 51]. The FAM134B-dependent ER-phagy is involved in CdTe-induced kidney injury [21]. However, whether FAM134B and its mediated ER-phagy are involved in tubular injury in DKD remains unknown. By searching the public database Nephroseq, we found that the mRNA levels of FAM134B were significantly downregulated in renal tubules of DKD patients, and we also demonstrated the down-regulation of FAM134B in DKD mice, implicating the involvement of FAM134B and its mediated ER-phagy in diabetic kidney injury. Besides, our research illustrated for the first time that in DKD mice, *Pacs-2* gene knockout inhibited FAM134B expression, suggesting an important role of PACS-2 in FAM134B regulation (Fig. 3 & Fig. 4). Taken together, these findings hinted that PACS-2 might contribute significantly in DKD tubular injury by activating ER-phagy through FAM134B.

FAM134B expression is known to be regulated by the transcription factor TFEB, which belongs to the microphthalmia-associated transcription factor/transcription factor E (MiTF/TFE) family and plays an important role in the maintenance of structural integrity and functional homeostasis in a variety of cells [52]. Recently, Cinque et al. also found that cytoplasmic TFEB enters the nucleus and binds to a CLEAR site in the third intron of the *Fam134b* gene to promote its transcription [36]. In the present study, we found that PACS-2 could reverse HG-inhibited TFEB nuclear translocation. PACS-2 is a membrane transporter protein [22]. The function of PACS-2 is derived from its distinctive structure. PACS-2 contains a cargo-binding region, a disordered intermediate region, and a carboxy-terminal region [22]. The cargo-binding region can bind to cargo proteins containing acidic clusters and transport these cargo proteins to corresponding organelles [22, 53–57]. In addition, the disordered intermediate region of PACS-2 contains a nuclear localization signal [22], suggesting that PACS-2 can carry cargo molecules to nucleus. In the current study, we found that TFEB contains typical acidic clusters that can be recognized by PACS-2. We also demonstrated that PACS-2 could bind to TFEB (Fig. 5). It suggested that PACS-2 may protect against tubulointerstitial inflammation and fibrosis in DKD through maintaining ER-phagy by interacting with TFEB and mediating its nuclear translocation.

**Fig. 6:**
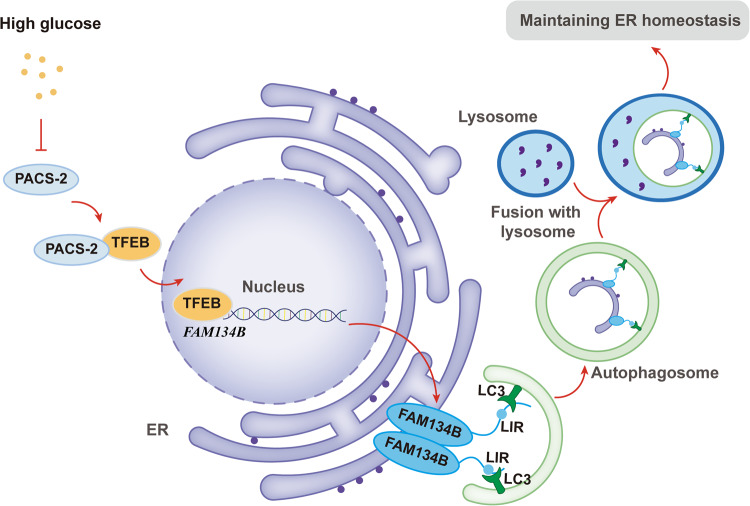
Schematic model summarizing our findings regarding the contribution of PACS-2 in ER-phagy and HG-induced renal tubular injury. PACS-2 is a membrane trafficking protein that promotes TFEB nuclear translocation, and thus inducing *Fam134b* gene transcription and facilitating ER-phagy process by interacting with LC3β. Under diabetic conditions, down-regulated PACS-2 decreases TFEB translocation and hence affects *Fam134b* gene transcription, followed by the decrease of the interaction between FAM134B and LC3β. ER-phagy is then blocked, leading to the accumulation of damaged or excessive ER in cytoplasm, and ultimately contributing to the activation of inflammatory response and the occurrence of fibrosis. LIR LC3-interacting region.

In the present study, we showed that HG downregulated the nuclear translocation of TFEB by inhibiting PACS-2 expression in tubular cells and then reducing its binding to TFEB, which in turn reduced *Fam134b* gene transcription and subsequently impaired ER-phagy, thus aggravating the imbalance of cellular homeostasis in renal tubules, the renal inflammatory cell infiltration, and the interstitial fibrosis (Fig. 6). Collectively, our findings suggested that PACS-2 deficiency may aggravate diabetic kidney injury through the TFEB/FAM134B/ER-phagy pathway.

### Supplementary information

Reproducibility checklist

Supplemental Material

## Data Availability

All data will be made available by the corresponding author upon reasonable request.
