# Supplementary material for: PACS-2 deficiency aggravates tubular injury in diabetic kidney disease by inhibiting ER-phagy
Source: Cell Death Dis. 2023 Oct 4;14(10):649. doi: 10.1038/s41419-023-06175-3 (PMC10550977; doi:10.1038/s41419-023-06175-3)

Supplementary Material

Full unedited western blots for Figure 1G

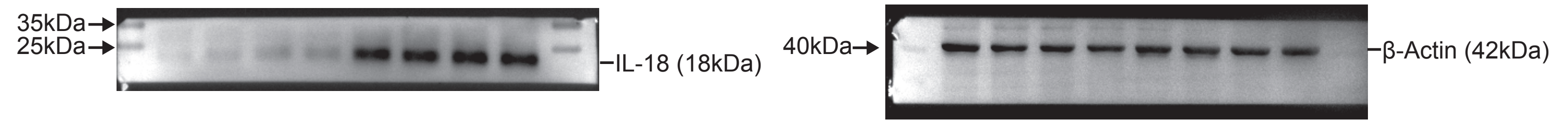

Full unedited western blots for Figure 1I

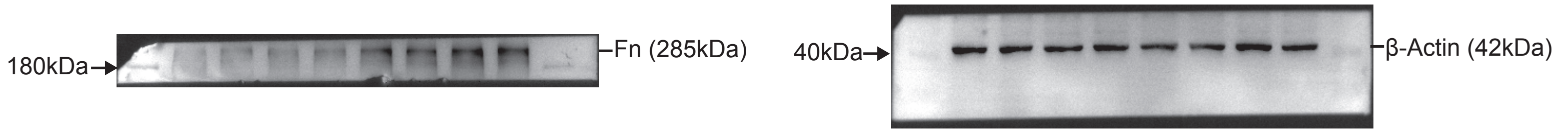

Full unedited western blots for Figure 2F

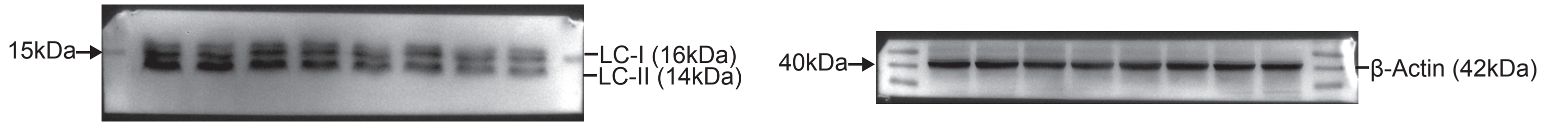

Full unedited western blots for Figure 2G

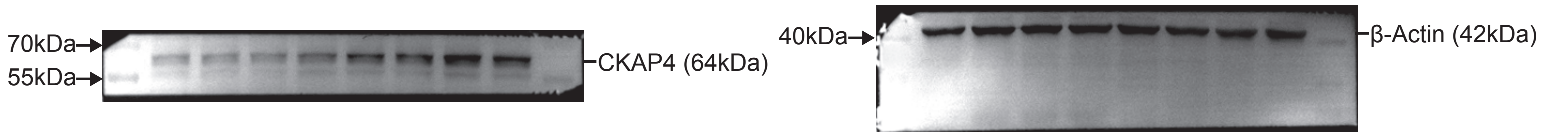

Full unedited western blots for Figure 3D

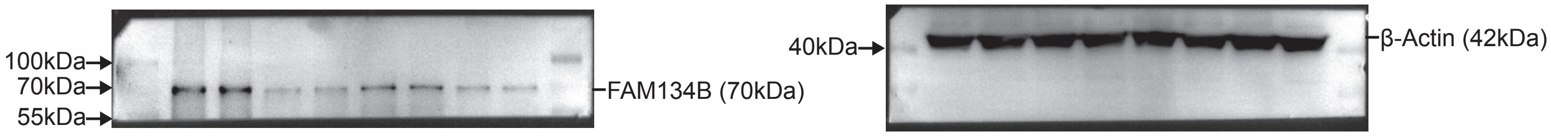

Full unedited western blots for Figure 4A

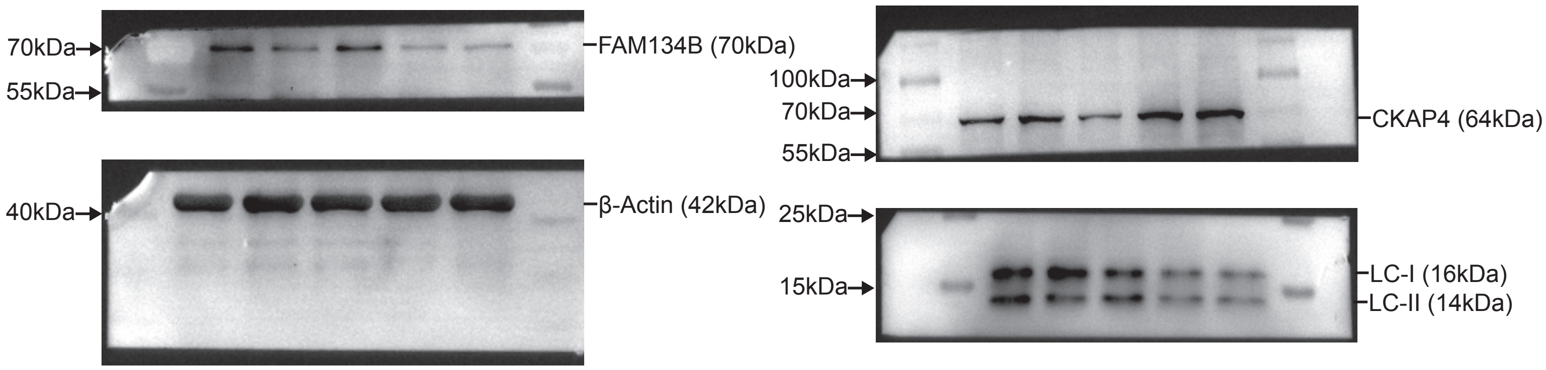

Full unedited western blots for Figure 4F

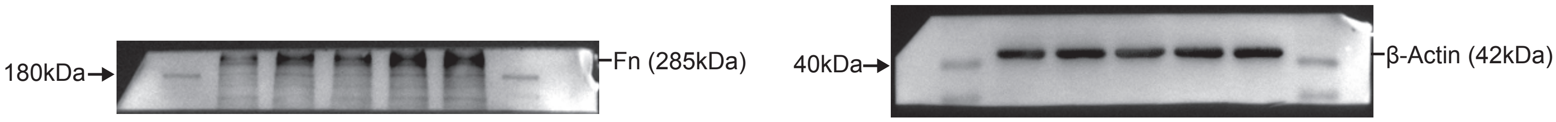

Full unedited western blots for Figure 5A

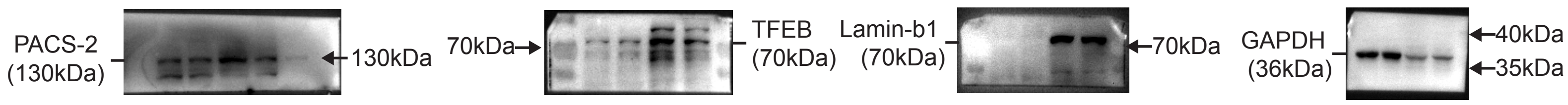

Full unedited western blots for Figure 5C

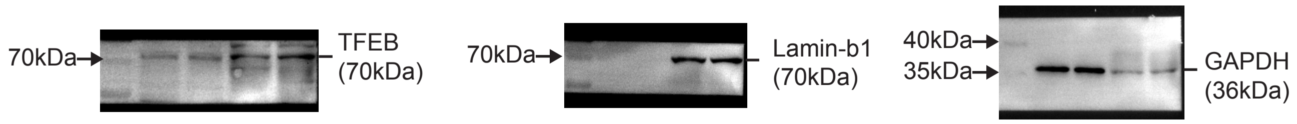

Full unedited western blots for Figure 5F

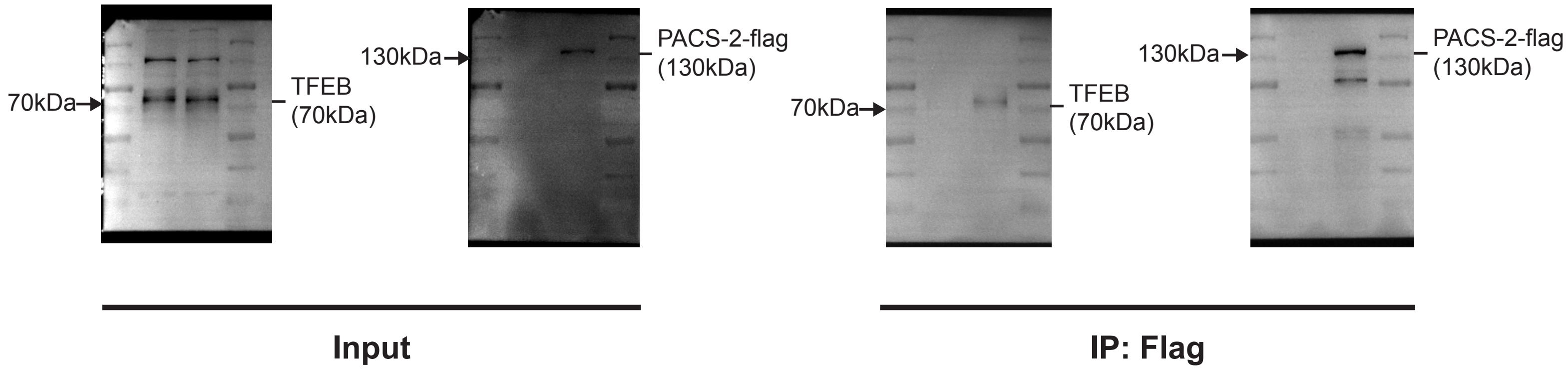

Supplement: Supplementary file 2 — Supplemental Material [file 41419_2023_6175_MOESM2_ESM.pdf]
